# The Impact of the Calibration Method on the Accuracy of Point Cloud Reconstruction from Underwater RGB and Multispectral Images

**DOI:** 10.3390/s26216871

**Published:** 2026-10-30

**Authors:** Paulina Kujawa, Jaroslaw Wajs, Damian Kasza

**Affiliations:** Department of Geodesy and Geoinformatics, Wrocław University of Science and Technology, Na Grobli 15, 50-421 Wroclaw, Poland; jaroslaw.wajs@pwr.edu.pl (J.W.); damian.kasza@pwr.edu.pl (D.K.)

**Keywords:** camera calibration, underwater photogrammetry, USV, shallow water, bathymetry

## Abstract

Accurate photogrammetric reconstruction of underwater areas requires careful selection of camera calibration strategies. However, evaluation of calibration methods for consumer-grade cameras in very shallow environments is limited. In this study, five calibration strategies and three configurations of Brown’s model parameters were evaluated for underwater 3D reconstruction. This was achieved using a GoPro Hero 12 RGB camera and a Mapir Survey3W multispectral camera mounted on an unmanned surface vehicle (USV), which was placed in a controlled laboratory tank with a maximum depth of 0.5 m. For the GoPro camera, the frame model outperformed the fisheye model in underwater conditions despite the use of a wide-angle lens. The reduced Brown model (omitting parameters *k*3, *k*4, *b*1 and *b*2) produced the smallest mean signed M3C2 distance relative to the UAV-derived reference point cloud (6 mm). For the Mapir camera, the full Brown model produced the smallest mean signed M3C2 distance (38 mm). In both cases, in situ auto-calibration provided comparable or the best accuracy across all configurations, eliminating the need to acquire separate underwater calibration targets. Aerial calibration was found to be unsuitable for both cameras, regardless of the parameter configuration. The optimal calibration strategy was found to depend on the type of camera, indicating that it cannot be assumed a priori.

## 1. Introduction

Mapping underwater areas is a field with great research and cognitive potential. A significant proportion of the population lives in coastal regions, so ensuring their safety poses a major challenge. Monitoring these areas enables changes to be detected, including morphological processes, as well as observations of flora and fauna. Another important aspect is adapting to climate change, including assessing its impact on the coastal environment and planning preventive measures in response to potential threats. Researchers have identified a wide range of applications for shallow-water mapping, including: marine habitat mapping [1,2], seagrass mapping [3,4], coral reef monitoring [5,6], detection and health assessment of underwater structures [7], and reconstruction of archaeological objects [8,9].

A variety of measurement technologies are currently used to monitor underwater areas. Traditional technologies rely on acoustic techniques and include single-beam and multibeam echo sounders [10], as well as sidescan sonar [11]. However, these techniques are better suited to mapping deeper underwater areas. In shallow waters, different approaches are used, which can be broadly divided into two categories: imaging- and LiDAR-based technologies [12,13]. Depending on the height of the platform relative to the water surface, data on shallow waters can be collected underwater (by divers [5], using remotely operated vehicles—ROVs [14]), from or near the water’s surface (using Unmanned Surface Vehicles—USV [15]), from low altitudes (using Unmanned Aerial Vehicles—UAV [16]), from medium altitudes (using aircrafts [17]) or from high orbital altitudes (using Satellites Derived Bathymetry—SDB [18]).

Recent advances in 3D sensing include geometry-aware depth completion for transparent and specular objects [19], colour-calibrated image reconstruction [20], and detection in underwater acoustic images [21]. Neural radiance fields adapted to underwater scenes [22] and neural networks for underwater camera calibration [23] are also active research directions [24]. Comparing point clouds from different platforms also requires accurate registration, for which learning-based methods have been proposed [25]. Furthermore, multi-platform surveys generate large volumes of 3D data, making the efficient storage, transmission and processing of point clouds an increasingly important issue [26].

Photogrammetric reconstruction relies on optical images, and underwater optical imaging is affected by wavelength-dependent light absorption, backscattering from suspended particles, uneven illumination, and low contrast [24,27,28]. These effects can hinder feature detection and matching in photogrammetric processing [24], and may therefore reduce the quality of camera calibration and 3D reconstruction. Image enhancement methods address these problems using diffusion models developed for low-light images [29], attention-based transformer networks for underwater images [30], adaptive enhancement [31], and joint enhancement and detection [32,33], but they were not used in this study. The short camera-to-object distance (0.5 m) used here limited the effects of light absorption and scattering [24].

Differences between imaging modalities and domains are widely studied in computer vision, for example in cross-modal matching [34], domain adaptation for object detection [35], and the transfer of knowledge between optical and SAR imagery [36]. The RGB and multispectral cameras used here differ in their imaging characteristics, so each was calibrated and processed separately.

Uncertainty in underwater sensing and navigation is addressed in several ways, including probabilistic prediction of 3D reconstruction quality [37], adaptive GNSS/INS positioning [38], and the perception of vehicle motion state [39]. However, these studies address tasks other than camera calibration. The following subsections review the calibration of the two types of consumer cameras used in this study.

### 1.1. Consumer GoPro Camera Calibration

Calibrating underwater cameras is a fundamental step in the photogrammetric processing of underwater images, enabling accurate 3D reconstruction. Carrying out this process underwater is considerably more difficult than in air. Refraction at the water–glass–air interface introduces systematic errors that must be corrected [40]. Selecting an appropriate model, geometry, and validation procedure is crucial for achieving accurate 3D models. Different calibration approaches are used depending on the type of optical port. Systems with a flat port introduce strong curvilinear distortions and optical aberrations [41]. The main approach involves using explicit refractive models based on ray tracing to optimise glass parameters. Examples of these models include Agrawal’s axial model and the Pinax model [42,43]. Implicit methods, on the other hand, treat refraction as a radial distortion but introduce systematic errors [44]. The accuracy of the calibration can be improved by applying empirical weighting to observations [41]. In the case of systems with a dome port, calibration is not required if the centre of the object’s perspective coincides with the centre of the dome’s curvature [45]. In practice, however, housing calibration is necessary to estimate the port’s eccentricity. Various methods are employed to compensate for refraction, including implicit methods using standard radial and decentering distortion polynomials [45], hybrid approaches combining in-air calibration with underwater housing parameters [46], and data-driven methods using neural networks [23]. The problem of refraction at the water–glass–air interface does not arise if cameras do not have an underwater housing. However, modelling the optical path still requires internal and external calibrations to be taken into account [47].

Advances in remote data acquisition are driving a shift towards the use of inexpensive, compact sensors, such as GoPro cameras, for collecting underwater data. The first studies to use GoPro cameras in an underwater environment were carried out to investigate the potential of using stereo cameras to take micro-bathymetric measurements of the seabed, and to create a point cloud with an average point spacing of approximately 3 mm and an accuracy of less than 1 cm [48].

Calibration is required when using camera images, including those from consumer cameras, for high-precision reconstruction. In [3], the authors precisely calibrated the intrinsic parameters of a GoPro Hero 2 camera (i.e., focal length, principal point, radial distortion and tangential distortion) using underwater recordings of a chessboard and the MATLAB Camera Calibration Toolbox (Bouguet). This method was also employed in a subsequent study [4], in which the authors calibrated a GoPro Hero 4 camera using a rigid, planar chessboard pattern. In [49], underwater measurements were carried out using a calibration frame. The influence of camera resolution and usage methods on calibration parameters was investigated.

In research presented in [50], the authors developed a system comprising four GoPro cameras mounted on a shared frame. They calibrated the cameras using underwater footage of a chessboard in order to obtain accurate intrinsic parameters, which are crucial given the optical changes that occur underwater. The authors tested the reconstruction with and without the calibrated intrinsic parameters, as well as with and without the rig constraint. They demonstrated that while calibrating the intrinsic parameters and using the rig constraint can affect reconstruction quality, good, accurate reconstruction can still be achieved with the four-camera system without prior calibration.

In [51], the researchers developed their own calibration framework for use in tests conducted both in the air and underwater. Two types of cameras were used: the Canon G7X and the GoPro Hero 5. Studies carried out showed poorer results for lower-end cameras. Nevertheless, the overall accuracy values obtained, expressed as a root mean square error of less than 2 mm, confirmed the effectiveness of the calibration frame in both aerial and underwater conditions.

The study by [52] involved developing a stereo vision system comprising two GoPro 11 cameras mounted on a micro-ROV. Calibration was performed in laboratory conditions (in air) and underwater. Different strategies were used to estimate the relative orientation of the cameras being tested. The results showed that the differences between calibrations performed in both environments were statistically insignificant, with the estimated baseline being practically the same length in both cases. The authors emphasised that calibration performed in air can be used effectively for underwater measurements.

Meanwhile, ref. [53] presented a system combining low-cost GoPro cameras with a ROV, additionally equipped with a lighting system, for the inspection of underwater hydropower infrastructure. The analyses were carried out by employing the following approaches to bundle block adjustment (BBA): (i) using ground control points (GCPs); (ii) not using GCPs but instead pre-calibrated camera parameters; and (iii) using both GCPs and pre-calibrated camera parameters. The latter approach achieved the smallest error values.

In summary, when using an imaging system for underwater photogrammetry, it is important to consider all components of the setup, including the camera, the housing port, and the surrounding aquatic medium. This is because light rays are refracted at boundaries between different media, such as between water and the port material (glass or acrylic), and between the port and the air–lens interface inside the housing. This phenomenon can be modelled using physical refractive models or ray tracing. However, this approach requires precise knowledge of the port geometry, its refractive indices, and the lens’s position relative to the housing. An alternative approach is to use classical calibration models, such as the Brown model. While these models do not describe refraction directly, they can partially compensate for its effect by adjusting the parameters for internal orientation and distortion. Models of this type are therefore useful in standard SfM procedures, but their parameters can depend heavily on acquisition conditions and be less stable when transferring between in-air calibration, underwater calibration, and real scene reconstruction. In the present study, the two cameras differed in their optical configuration: the GoPro Hero 12 was deployed without a housing, with refraction occurring only at the single water–lens interface, whereas the Mapir Survey3W was enclosed in a plastic housing, introducing an additional refractive boundary between water and the housing material.

### 1.2. Consumer Multispectral Camera Calibration

In underwater applications, multispectral cameras are primarily used for seabed mapping, monitoring ecosystems, improving material discrimination and identifying archaeological objects [54,55]. However, their use is limited by strong wavelength-dependent absorption and scattering, as well as calibration difficulties [56,57]. Compared with RGB cameras, spectral systems provide more radiometric and material information. This is particularly important when conventional images lose contrast and colour, making it impossible to identify objects.

One area of development in 3D underwater reconstruction methods is multispectral underwater stereophotometry. This builds on classical photometric stereophotometry by incorporating data from multiple spectral bands. In their research, Jiao et al. [58], proposed a method for the high-resolution 3D reconstruction of underwater objects based on combining depth data with multispectral stereophotometric analysis. This study focused on reconstructing the geometry of underwater objects using a single RGB-D image. The proposed approach involved pre-processing the image, including backscatter removal and image segmentation using the Simple Linear Iterative Clustering (SLIC) method. Additionally, low-frequency depth information was combined with high-frequency surface details obtained via multispectral stereophotometry. This ultimately led to an increase in the accuracy of the object’s geometric reconstruction. The paper by Yang et al. [59] presents a method for 3D underwater reconstruction that combines laser triangulation and multispectral stereophotometry. Multispectral imaging enables detailed surface geometry information to be obtained from a single image, while laser triangulation provides information about the object’s actual scale. Integrating these two techniques produced reconstructions that were denser and more accurate than those obtained using either method separately.

Multispectral cameras require separate geometric and radiometric calibrations. Radiometric correction involves converting digital numbers (DN) to radiance and then to the object’s reflectance, taking into account the effects of immersion and the water column [60]. The underwater environment is more challenging than the atmospheric environment due to the immersion of the optical system, wavelength-dependent absorption and scattering, and path radiance [61].

Despite the growing interest in multispectral imaging for underwater applications, consumer-grade multispectral cameras such as the Mapir Survey3W RGN (Red+Green+NIR) have been employed primarily in aerial (UAV-based) configurations for above-water mapping [62].

Although the calibration of GoPro cameras has been studied in air and underwater, the choice of camera model and distortion parameters for direct deployment in very shallow water has not been systematically compared. No research into the direct underwater deployment of consumer multispectral cameras for image-based 3D reconstruction was found. In this study, the authors conducted an experiment in a shallow-water reservoir that had been constructed artificially. RGB and multispectral cameras were mounted on a USV and submerged below the surface of the water to collect underwater imagery. Meanwhile, RGB data acquired by a UAV was used to validate the results. The aim was to evaluate the impact of the calibration strategy and the configuration of the Brown distortion model on the accuracy of underwater photogrammetric reconstruction.

### 1.3. Contributions

This study makes the following contributions to underwater image photogrammetry:

(1) A systematic evaluation of five calibration variants for two types of cameras, an action RGB camera-GoPro Hero 12 (GoPro, Inc., San Mateo, CA, USA) and a multispectral camera-Mapir Survey3W (MAPIR, Inc., San Diego, CA, USA), in a controlled, very shallow water environment (depth ≤ 0.5 m), which has rarely been analysed in the literature to date.
(2) A quantitative evaluation of the effect of the selected camera model (fisheye or frame) on the accuracy of underwater photogrammetric reconstruction.
(3) A comparison of the three variants of the Brown model parameterisation (reduced, extended and full) for all five calibration strategies, validated against UAV point clouds using the M3C2 method, and compared with direct GNSS profile measurements using the C2C method.

## 2. Materials and Methods

### 2.1. Study Site and Data Acquisition

**Figure 1:**
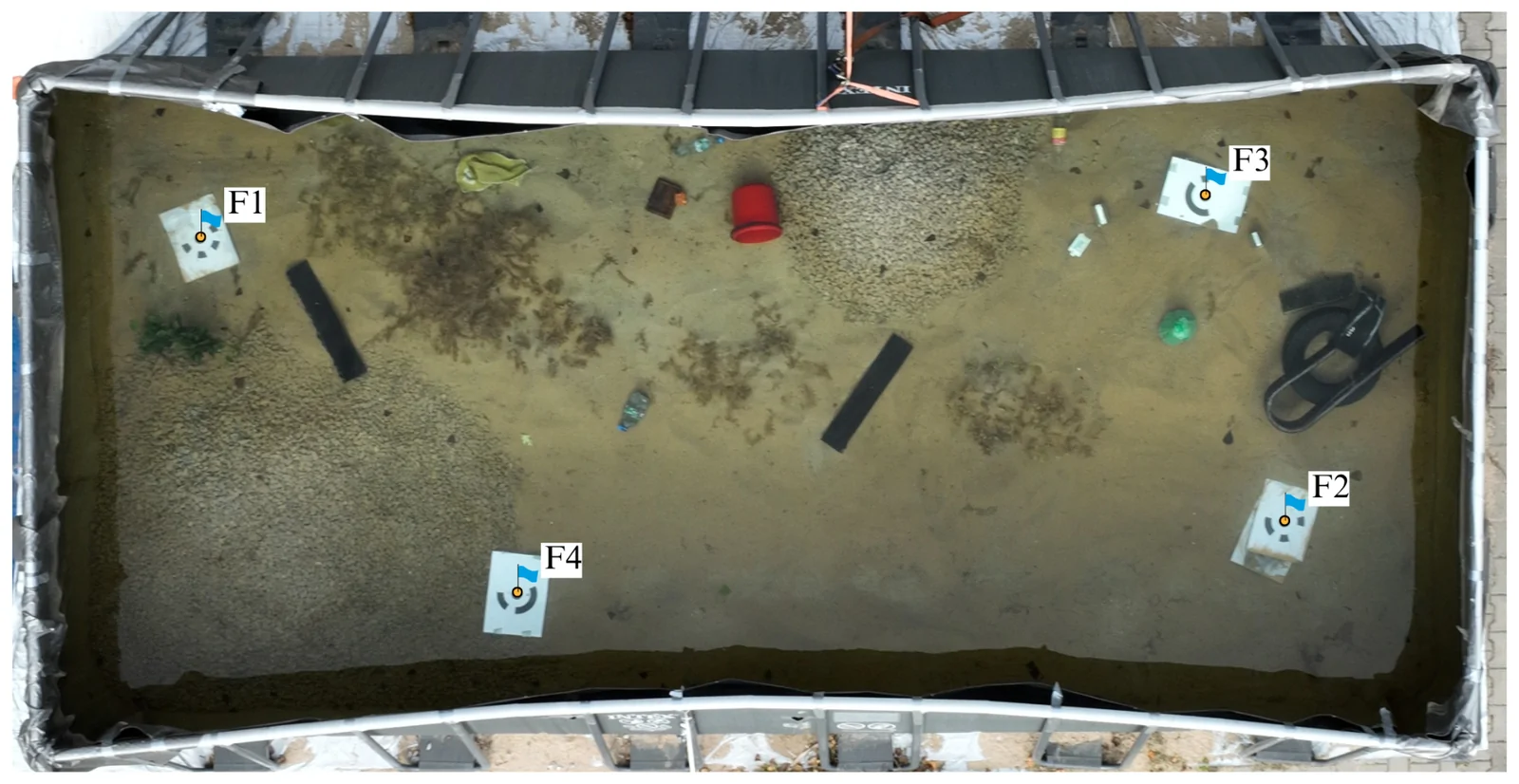
An experimental study site with 4 underwater GCPs.

The experiments were conducted on a dedicated test site. A water reservoir measuring 3.7 × 7 m and approximately 0.6 m in depth was constructed to simulate shallow-water conditions. The bottom of the reservoir was prepared using sand, gravel, vegetation, and other materials of various textures and colors. For georeferencing and validation purposes, four underwater control points were established within the reservoir. Their coordinates were measured using the GNSS RTK technique with a Leica GS18 T receiver (Leica Geosystems AG, Heerbrugg, Switzerland), providing an accuracy of 8 mm + 0.5 ppm in the horizontal component and 15 mm + 0.5 ppm in the vertical component (Network RTK). All four points were used as control points, and no independent check points were used. The research site with GCPs is shown in Figure 1.

**Figure 2:**
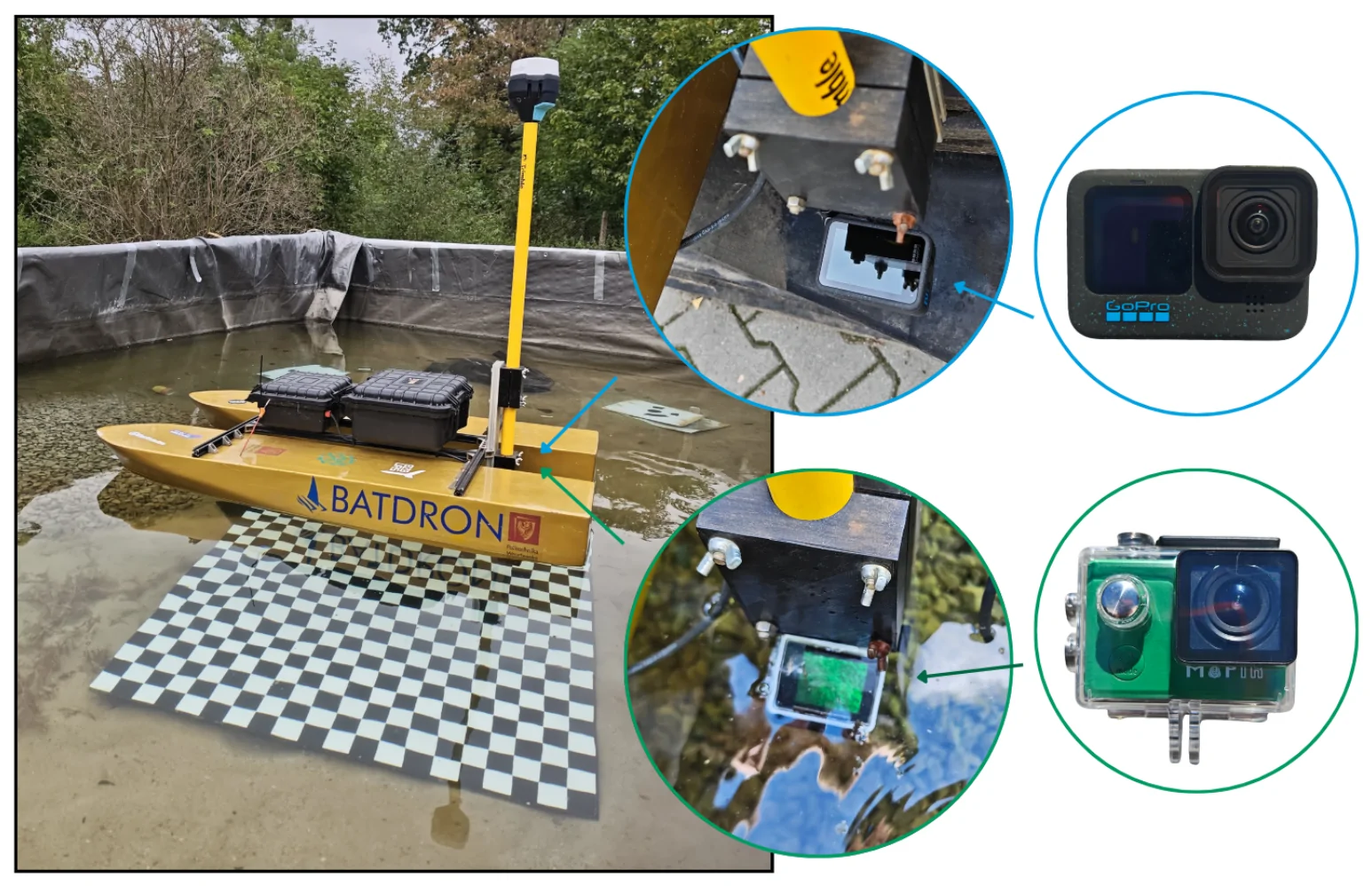
Underwater image acquisition system: USV equipped with a GoPro/Mapir camera during calibration panel image acquisition.

Images for photogrammetric purposes were gathered using two interchangeable cameras mounted on a partially submerged USV platform [63] (Figure 2). Detailed technical specifications of the cameras are provided in Table 1. The first series of experiments included taking photos of a 1.5 × 1 m calibration panel both in air and underwater. In the second series, underwater photos of the reservoir bottom were collected.

### 2.2. Reference Datasets

UAV surveys were also conducted using a DJI Mavic 3M drone (SZ DJI Technology Co., Ltd., Shenzhen, China) flying at an altitude of 12 m. The onboard RGB camera captured images at a resolution of 5280 × 3956 pixels (20 MP). Six terrestrial GCPs were used to georeference the UAV data, while the four underwater GCPs were used as check points. Their coordinates were measured using a Trimble S7 total station (Trimble Inc., Westminster, CO, USA), with an angular accuracy of 5″ and a distance accuracy of 1.0 mm + 2 ppm.

The UAV data were used as an external reference to evaluate the geometric agreement of the underwater reconstructions. Both the UAV- and USV-derived point clouds were georeferenced independently in a common geodetic reference frame.

In addition, two measurement profiles were taken inside the reservoir using GNSS RTK technology. These profiles included more than a dozen measurement points. They were then used to independently validate the reconstructed reservoir bottom surface using various data processing methods.

The accuracy of the refraction-corrected UAV point cloud was assessed by comparing it with GNSS-measured profiles. The mean 3D distance was 16 mm, with a median of 15 mm and a standard deviation of 10 mm. The mean vertical offset was −14 mm. The C2C differences also showed a spatial gradient across the study area, ranging from approximately −39 mm to +5 mm. This pattern may be related to residual errors in the refraction correction and the configuration of the GCP network.

### 2.3. Concept of Data Processing

**Figure 3:**
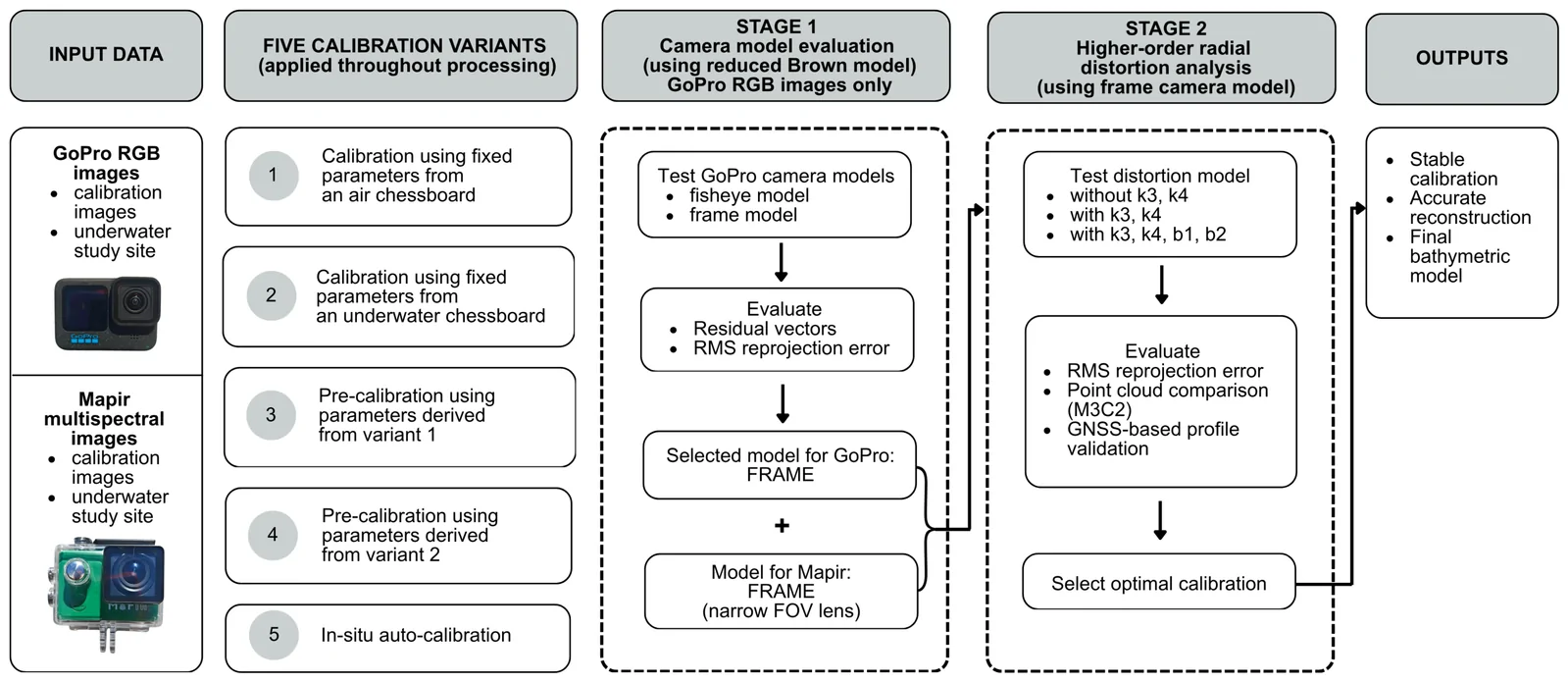
Experimental workflow for underwater camera calibration and reconstruction accuracy assessment.

The calibration procedure (Figure 3) was developed to evaluate how the choice of GoPro camera model and the complexity of the calibration model impact the quality of underwater reconstructions based on RGB and multispectral images.

Five processing approaches were used to investigate the stability and transferability of underwater camera calibration for the GoPro and Mapir cameras. These strategies correspond to: Variant 1—calibration using fixed parameters from an air chessboard; Variant 2—calibration using fixed parameters from an underwater chessboard; Variant 3—pre-calibration using parameters derived from Variant 1; Variant 4—pre-calibration using parameters derived from Variant 2; and Variant 5—in-situ auto-calibration.

All photogrammetric data processing was conducted in Agisoft Metashape Professional (v. 2.2.2) [64,65,66]. Two camera types available in Metashape were used: “Frame” and “Fisheye” [67]. The “Frame” type is a central perspective (pinhole) model with Brown-Conrady lens distortion [68]. The “Fisheye” type uses a fisheye projection with a polynomial distortion, and its radial part has the same form as in the Kannala–Brandt model [69]. The following parameters can be estimated: [67]: *f*—focal length (in pixels); cx,cy—principal point offset (in pixels); k1,k2,k3,k4—radial distortion coefficients (dimensionless); p1,p2—tangential distortion coefficients (dimensionless); and b1,b2—affinity and non-orthogonality (skew) coefficients (in pixels).

The processing strategy involved the following steps:

(i) **Camera calibration evaluation.** Two camera models (fisheye and frame) were tested for the GoPro images. In both cases, calibration was initially performed using a reduced set of Brown model parameters (f,cx,cy,k1,k2,p1,p2). The higher-order coefficients (k3,k4) and the affine parameters (b1,b2), analysed in stage 2, were deliberately omitted. This approach allows the influence of the camera model selection to be isolated from the influence of the calibration parameter configuration. The suitability of each model was evaluated based on the residual vector distributions and RMSE reprojection errors. The frame model was adopted as the standard choice corresponding to the optical characteristics of the Mapir multispectral camera’s narrow-angle lens.
(ii) **Assessment of higher-order radial distortion coefficients.** After selecting a frame model for the GoPro camera and adopting the same model for the Mapir camera, additional tests were conducted with and without the higher-order radial distortion coefficients (k3,k4), as well as affinity and non-orthogonality coefficients (b1,b2). Since underwater images taken with a wide-angle lens can still show complex radial distortions, particularly at the edges of the image, the influence of these parameters was examined further. The final results were presented as dense point clouds and compared with point clouds obtained from UAV data to which refractive correction had been applied. This was achieved by calculating cloud-to-cloud distances and analysing the basic statistics of these distances. Additionally, visualisations were prepared to illustrate the spatial distribution of distance differences. Furthermore, the distances between the point clouds and the profile points from GNSS were determined. A comparison with the UAV reference cloud was performed using the M3C2 method. However, due to the nature of the reference data, validation against point-based GNSS measurements was carried out using the C2C method. Finally, the relevant statistical measures were calculated based on these distances.

Although refraction at the water–lens interface was not explicitly modelled, it was indirectly compensated for through the parameters of the Brown model, which were determined during the calibration process. While this simplification may result in residual systematic errors, these residual effects are reflected in the external validation metrics based on the M3C2 and C2C comparisons. The M3C2 distances were calculated in CloudCompare (v. 2.13.2) [70] using the drone point cloud as the reference and the underwater point cloud as the comparison. The normal scale and projection scale were both set to 0.05 m, and the maximum depth of the cylinder was set to 0.40 m. The normals were oriented in the +Z direction. Distances were calculated for all points. No radiometric correction was performed, since the study aimed to evaluate the geometric accuracy of the reconstruction rather than analyse the data spectrally or in terms of colour. However, such a correction would be necessary for further multispectral or reflectance interpretation [71].

## 3. Results

### 3.1. RGB GoPro Camera

#### 3.1.1. Camera Model Selection

**Figure 4:**
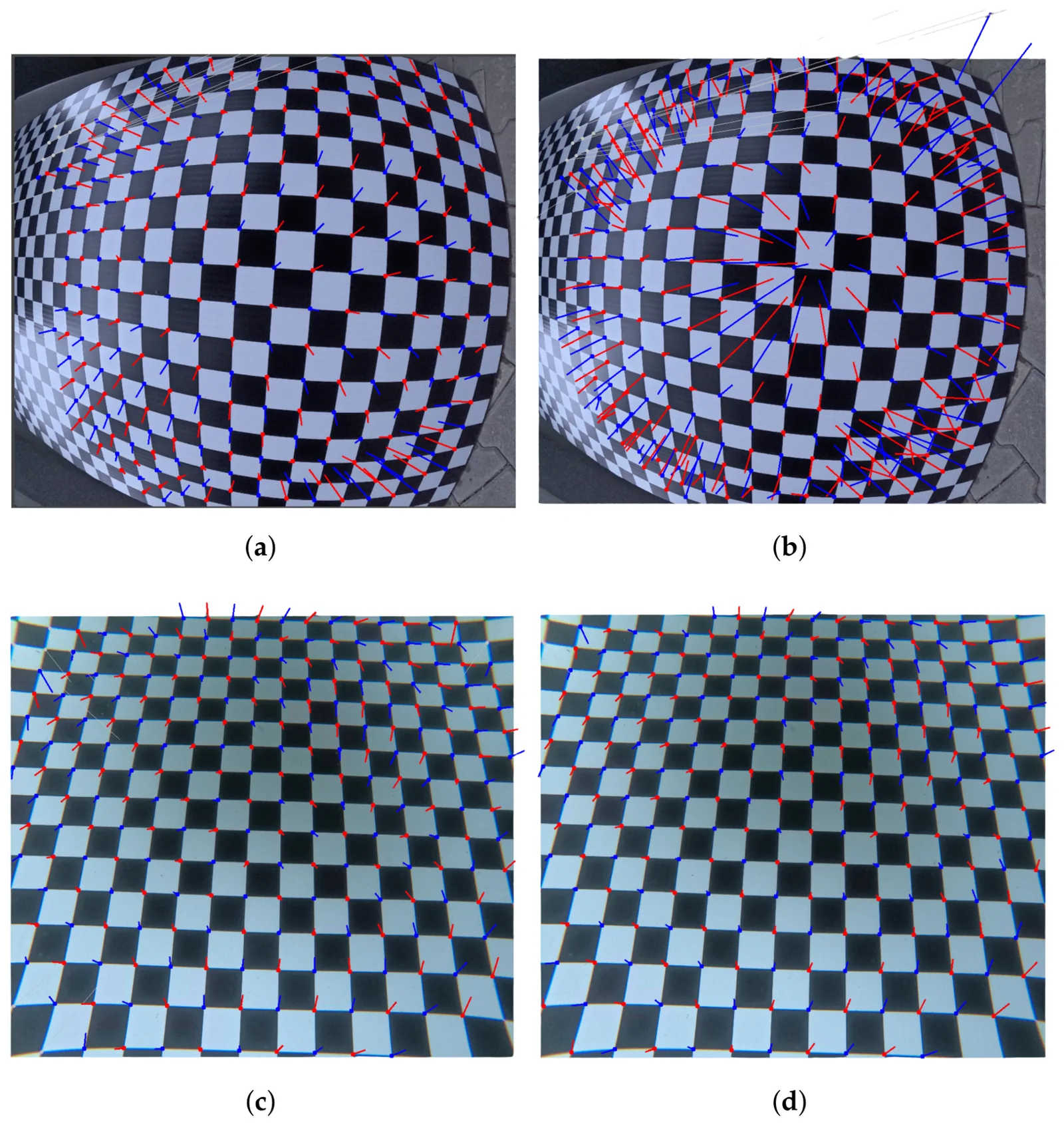
Image residual vectors for the calibration chessboard capture with the GoPro: (**a**) in air with fisheye lens, (**b**) in air with frame lens, (**c**) underwater with fisheye lens, and (**d**) underwater with frame lens.

Despite using a GoPro camera in wide-angle mode, the short shooting distance (approximately 0.5 m) and the lens’s different optical characteristics in an aquatic environment compared to in the air may have affected the geometry of image distortion. Consequently, the suitability of the available camera models in the Metashape software, the fisheye and frame models, for underwater reconstruction was assessed. The camera model was evaluated using a reduced set of Brown model parameters. Figure 4 illustrates the residual displacement vectors for the detected control points in the selected representative images obtained following calibration in Agisoft Metashape. It illustrates how the fit of the camera model depends on the acquisition environment (air or water) and the camera model used. For images taken in the air, the fisheye model generates small, evenly distributed residual vectors (Figure 4a), indicating a good fit to the GoPro lens’s optical characteristics in this environment. In contrast, applying the frame model to the same images (Figure 4b) results in larger residual vectors in the peripheral areas, indicating difficulties in capturing extensive barrel distortion. Under underwater conditions, the chessboard image exhibits notably less geometric distortion. Both the fisheye model (Figure 4c) and the frame model (Figure 4d) produce visually similar residual vector distributions.

Photogrammetric processing was then carried out on five variants of underwater reservoir images. The root mean square error (RMSE) results for reprojecting both camera models are summarised in Table 2. The RMSE values for Variant 1 were extremely high for both models. This confirms the unsuitability of calibration parameters determined in the air for direct use in underwater reconstruction without re-optimisation. Excluding Variant 1 as an outlier, the fisheye model achieves RMSE values ranging from 0.89 to 1.93 pixels, while the frame model achieves values ranging from 0.27 to 1.11 pixels. In variants based on underwater calibration and in-situ autocalibration, the frame model consistently achieves lower RMSE values than the fisheye model. This represents an improvement factor of between 3.6 and 5.3. The only exception is Variant 3, where the fisheye model’s slight advantage is likely the result of it inheriting incorrect initial parameters from Variant 1.

The calibration parameter values for all variants are shown in Table A1. The fisheye model parameters demonstrate considerable variation between variants, suggesting calibration instability. The focal length (*F*) ranges from 2448 to 6134 pixels, which is physically implausible for a fixed-focal-length lens and indicates a 2.5-fold range. The k1 coefficient changes sign between variants (from +0.0408 to −0.1263), while the k2 coefficient ranges from 0.0024 to 1.8070. This suggests that these parameters may compensate for systematic modelling errors rather than describing actual optical distortions. By contrast, the frame model parameters exhibit lower variability: the focal length ranges from 2316 to 3789 pixels (a 1.6-fold range); k1 consistently takes on negative values (from −0.0989 to −0.1660); and k2 remains consistent in sign and magnitude (from 0.0173 to 0.1775). The frame model’s parameters are more stable and have lower reprojection RMSE values. This confirms that the frame model better fits the optical characteristics of the GoPro lens in underwater conditions.

Despite the use of a wide-angle lens, an analysis of the reprojection RMSE and the variability in the calibration parameters clearly indicates the superiority of the frame model in underwater conditions. This model was therefore selected for further processing.

#### 3.1.2. Higher-Order Radial Distortion Analysis

After selecting the frame model in Stage 1, the analysis examined the impact of higher-order radial distortion coefficients on the accuracy of underwater photogrammetric reconstruction using a GoPro Hero 12 camera. During the camera model assessment, Brown’s reduced model (f,cx,cy,k1,k2,p1,p2) yielded satisfactory reprojection residuals. However, wide-angle lenses used in underwater environments may show complex radial distortion. These patterns are particularly noticeable at the edges of the image and cannot be fully described by lower-order coefficients. To investigate this, three parameter setups were tested for all five processing variants: the reduced model (without k3,k4,b1 and b2), the extended model (with k3 and k4), and the full model (with k3,k4,b1 and b2). The calibration parameter values for the GoPro camera are presented in Table A2. The configurations were evaluated based on reprojection errors, a comparison of dense point clouds with UAV reference data, and GNSS profile validation.

In Variant 1, image registration failed due to high reprojection errors. This resulted in the incorrect placement of GCPs, erroneous estimates of camera positions, and incorrect geometry of the sparse point cloud. Variant 1 was therefore excluded from further processing. Four underwater GCPs were used in the photogrammetric processing. The errors in determining their coordinates are presented in Table 3.

For the reduced configuration, Variants 2 and 4 exhibit higher total errors than Variants 3 and 5. This is due to the large Z-coordinate errors. The extended configuration (without b1 and b2) shows an improvement in Z error for Variants 3–5. However, this leads to increased image error. This may indicate partial overfitting of the model. The full configuration (with k3,k4,b1 and b2) yields the best results for Variants 4 and 5. However, this improvement is selective, as Variants 2 and 3 do not show a similar reduction. This suggests that parameters b1 and b2 only stabilise calibration when combined with a suitable starting point for optimisation.

**Figure 5:**
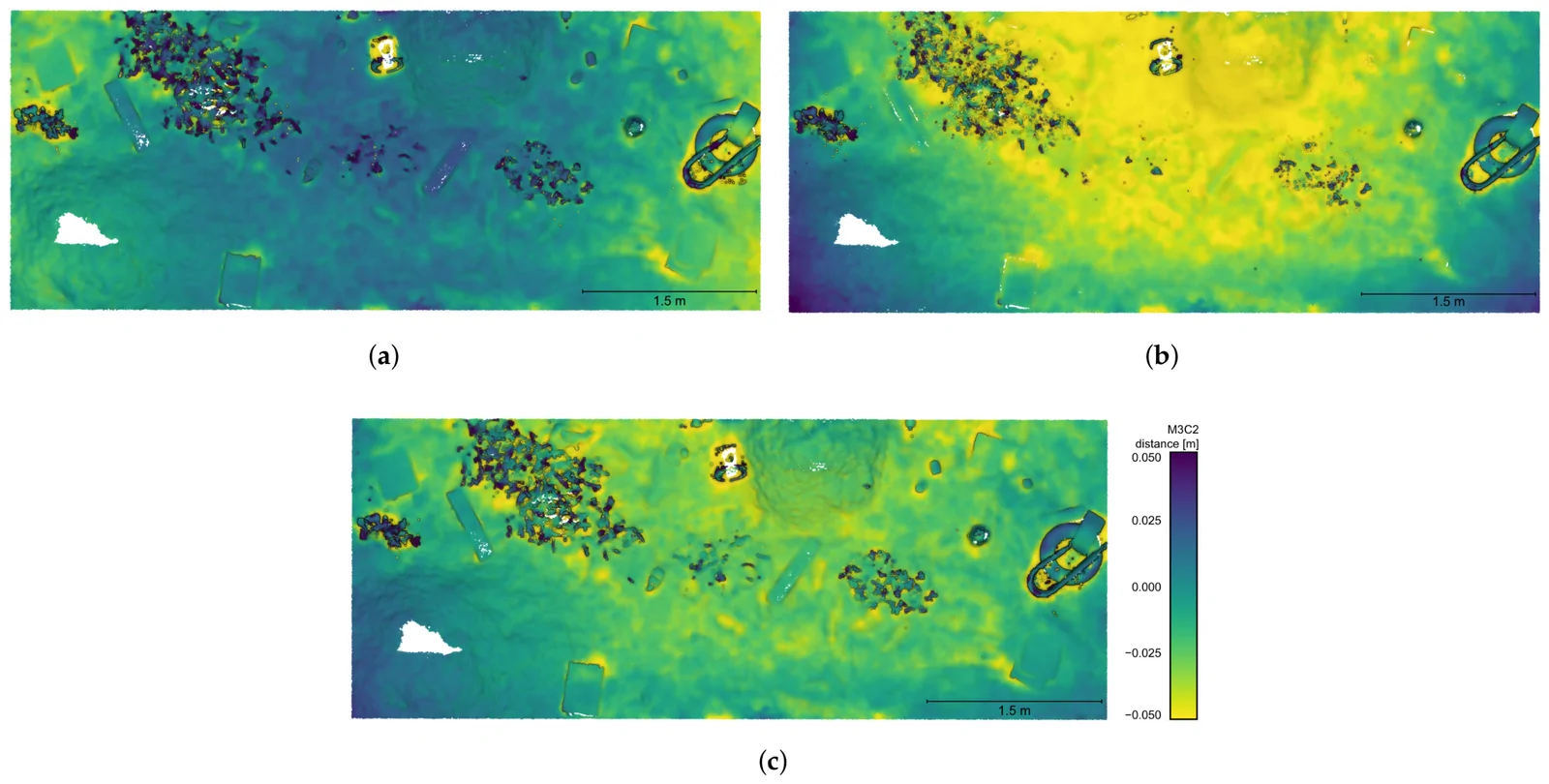
Spatial distribution of M3C2 distances for GoPro Variant 5 relative to the UAV-derived reference point cloud for three Brown model parameter configurations: (**a**) reduced model, (**b**) extended model, and (**c**) full model.

The distance statistics for M3C2 relative to the UAV reference point cloud are presented in Table 4, and the spatial distribution of distances for Variant 5 is illustrated in Figure 5. In all configurations, Variant 2 exhibits the largest M3C2 deviations relative to the UAV-derived reference point cloud, confirming the limited transferability of fixed underwater calibration parameters to scene reconstruction. Among all configurations, Variants 3 and 5 show the smallest magnitudes of the mean signed M3C2 distance in the reduced configuration. Variant 4 exhibits a negative mean 3D value, indicating a systematic underestimation of the reconstruction elevation. The M3C2 map (Figure 5a) corroborates this finding. The figure is dominated by values close to zero, with local deviations concentrated on objects, and the flat bottom of the reservoir is mapped with high spatial evenness.

**Figure 6:**
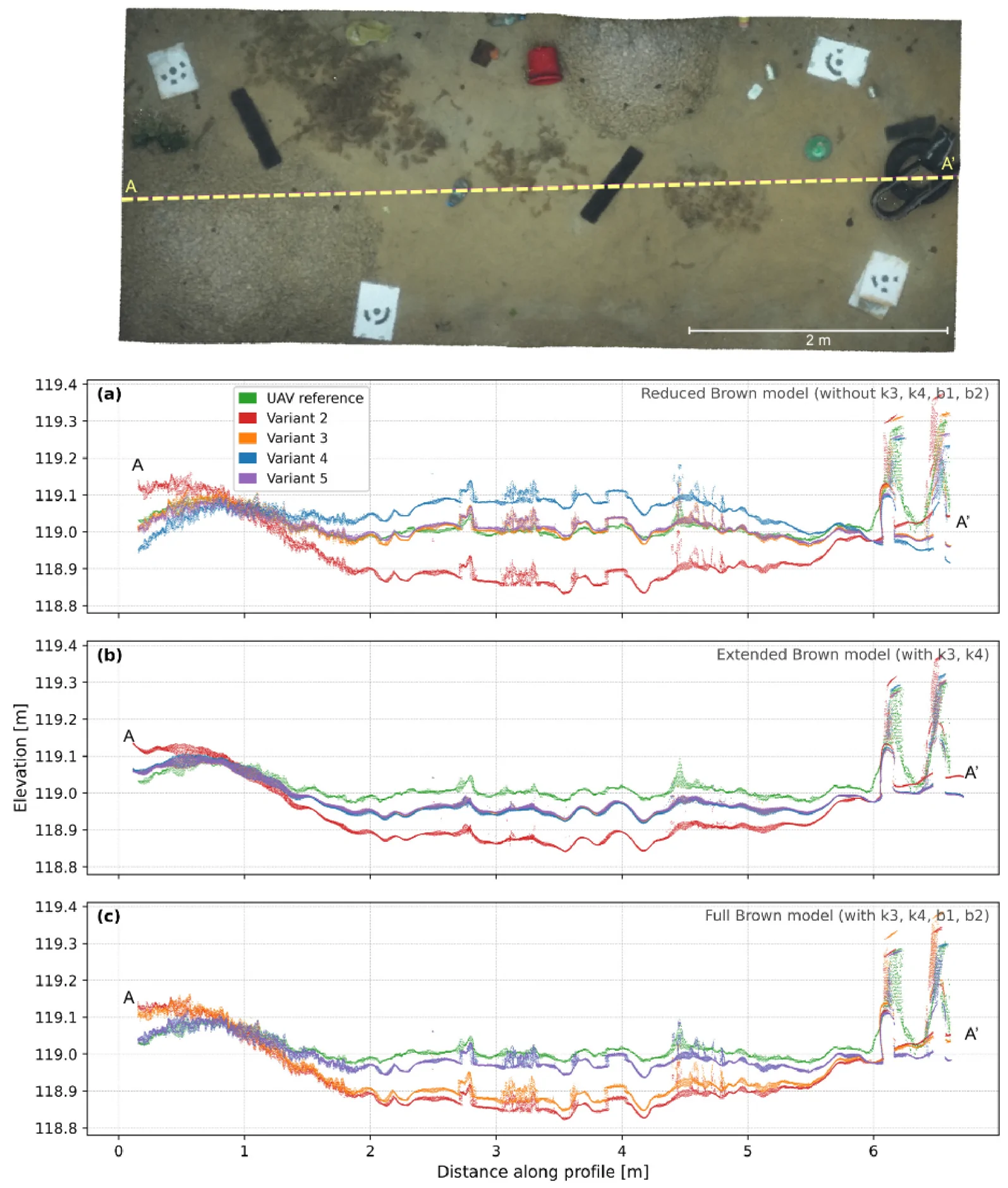
Cross-sectional profiles along transect A–A′ for three Brown model configurations: (**a**) reduced model, (**b**) extended model, and (**c**) full model.

A visual analysis of the cross-sections (Figure 6) confirms the quantitative results in Table 3 and Table 4. In all configurations, Variant 2 systematically underestimates elevation relative to the UAV reference. For the reduced configuration, Variant 4 shows a clear overestimation of elevation between 1 and 5.5 m, whereas Variants 3 and 5 align almost perfectly with the reference. The extended configuration eliminates the deviation of Variant 4. However, Variants 3–5 all exhibit a consistent shift relative to the UAV along the entire profile, which is consistent with the spatial variation visible on the M3C2 map (Figure 5). This indicates the presence of a systematic error introduced by k3 and k4. In the full configuration, Variants 4 and 5 achieve high agreement with the reference, with a deviation of no more than a dozen millimetres or so. In contrast, Variants 2 and 3 exhibit considerable deviations. This demonstrates that b1 and b2 only stabilise the reconstruction when the optimisation begins from the correct initial point.

The validation results for GNSS profiles (Table 5) are consistent with M3C2 vs. UAV observations. In all configurations, Variant 2 exhibits the highest mean 3D values. This reaffirms the limited transferability of fixed underwater calibration parameters. For the reduced configuration, Variants 3 and 5 produce similar results, whereas Variant 4 shows higher errors due to a substantial systematic offset in the Z component. Adding coefficients k3 and k4 does not clearly improve the results. While Variant 4 shows a reduction in error, Variants 3 and 5 show slight deterioration. The full configuration yields the best results for Variants 4 and 5.

For Variant 5, the extended configuration had a lower total GCP error than the reduced configuration (9 mm vs. 12 mm), but a higher image error (1.224 px vs. 0.710 px) and a larger mean M3C2 distance relative to the UAV reference (29 mm vs. 6 mm). The full configuration achieved both a lower image error (0.365 px) and a lower total GCP error (5 mm), but its mean M3C2 distance was still higher than that of the reduced configuration (21 mm vs. 6 mm). These results show that lower reprojection errors and GCP residuals do not necessarily translate into better agreement with the external reference datasets in object space, as they reflect the internal fit of the bundle adjustment. Therefore, the preferred configuration for the present dataset was identified based on the M3C2 and C2C comparisons with the external reference datasets.

To support the interpretation of differences between calibration configurations, 95% confidence intervals for mean M3C2 and C2C distances were computed. All values are reported in Tables 4, 5, 8 and 9. The intervals describe only the sampling uncertainty of the mean values. Differences between strategies with overlapping intervals are treated as equivalent. Differences of a few millimetres are not interpreted as meaningful, because they are smaller than the uncertainty of the reference data.

### 3.2. Multispectral Mapir Camera

As with the GoPro camera, calibration images of the checkerboard panel were taken from a distance of around 0.5 m, both above and below the water’s surface. These image sets were used to determine the internal parameters of the Mapir Survey3W multispectral cameras for five processing variants. Due to the narrow field of view of the Mapir camera lens, no comparison with the fisheye model was performed. The RMSE values for the reprojection of all variants are presented in Table 6. Variant 1 achieves extremely high values, comparable with those obtained using a GoPro camera. This once again confirms that calibration parameters determined in air are not suitable for direct use in underwater reconstruction. Variants 2–5 exhibit consistent calibration results, with Variants 3–5 yielding identical RMSE values within the present dataset. This consistency is greater than that observed for the GoPro camera and may be related to the lower optical distortion of the Mapir camera’s narrow-angle lens.

The calibration parameter values for the Mapir camera are presented in Table A3. The frame model parameters demonstrate high stability across variants. The focal length (*F*) remains almost identical across all configurations. The coefficients k1,k2,p1 and p2 are consistent in sign and magnitude across all configurations. Variant 2 is characterised by slightly greater variability in the parameters, particularly for k2 in the extended and full configurations. Variant 1 shows clearly different parameter values from the remaining variants. In particular, its *k*1 coefficient is close to zero compared with Variants 3–5. The high consistency of the higher-order parameters (k3,k4,b1 and b2) across Variants 3–5 confirms that the calibration results are repeatable and independent of the parameter configuration.

Four underwater GCPs were used in the photogrammetric processing. The positioning errors for the three configurations of Brown’s model parameters are presented in Table 7. Variant 1 was excluded from further processing due to its high total and image error values. Variants 3–5 exhibit high stability and almost identical total errors across all three configurations. This is a significant difference compared to the GoPro camera, where results varied more widely between variants. Variant 2 exhibits higher total errors in the reduced and full configurations, whereas the error reduces in the extended configuration. In Variant 2, the Z-component error accounted for the largest proportion of the total error. This suggests that there are limitations in using fixed calibration parameters, as determined on the calibration panel, to reconstruct the actual scene. No significant differences in GCP error values were found when comparing the three parameter configurations used in Variants 3–5. These results imply that, in contrast to the GoPro camera, adding higher-order coefficients does not enhance the accuracy of control point determination with the Mapir camera.

The statistics for the M3C2 distances relative to the UAV reference point cloud for the Mapir camera are presented in Table 8. Variant 2 achieves the highest mean 3D values across all configurations, and these values remain virtually identical regardless of the parameter configuration. Variants 3–5 demonstrate high consistency with each other within each configuration. The reduced and extended configurations yield nearly identical results, indicating that adding the k3 and k4 coefficients did not reduce the M3C2 deviations for the Mapir reconstruction in the present dataset. The full configuration produced slightly smaller mean signed M3C2 distances.

**Figure 7:**
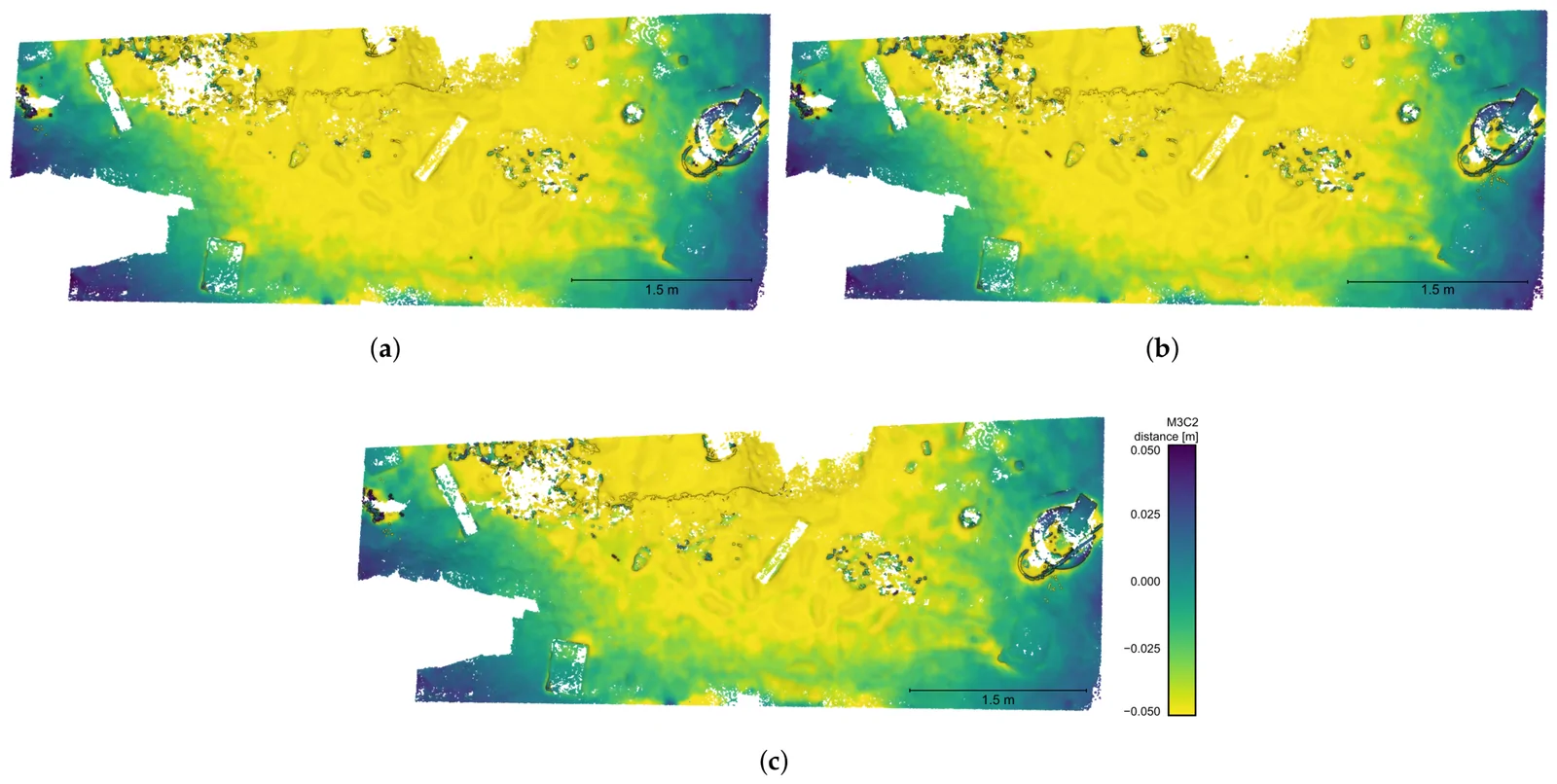
Spatial distribution of M3C2 distances for Mapir Variant 5 relative to the UAV-derived reference point cloud for three Brown model parameter configurations: (**a**) reduced model, (**b**) extended model, and (**c**) full model.

The spatial distribution of M3C2 distances for the Mapir camera configuration (Figure 7) shows a spatial gradient across all three configurations. Lower values are concentrated in the left part of the area, while values close to the maximum scale are concentrated in the right part. The reduced and extended configurations show nearly identical spatial distributions (Figure 7a,b). This confirms that adding the *k*3 and *k*4 coefficients does not affect the geometry of Mapir camera reconstruction. However, the full configuration (Figure 7c) only yields a partial improvement in the left part of the area, with consistent deviations visible along the entire profile.

**Figure 8:**
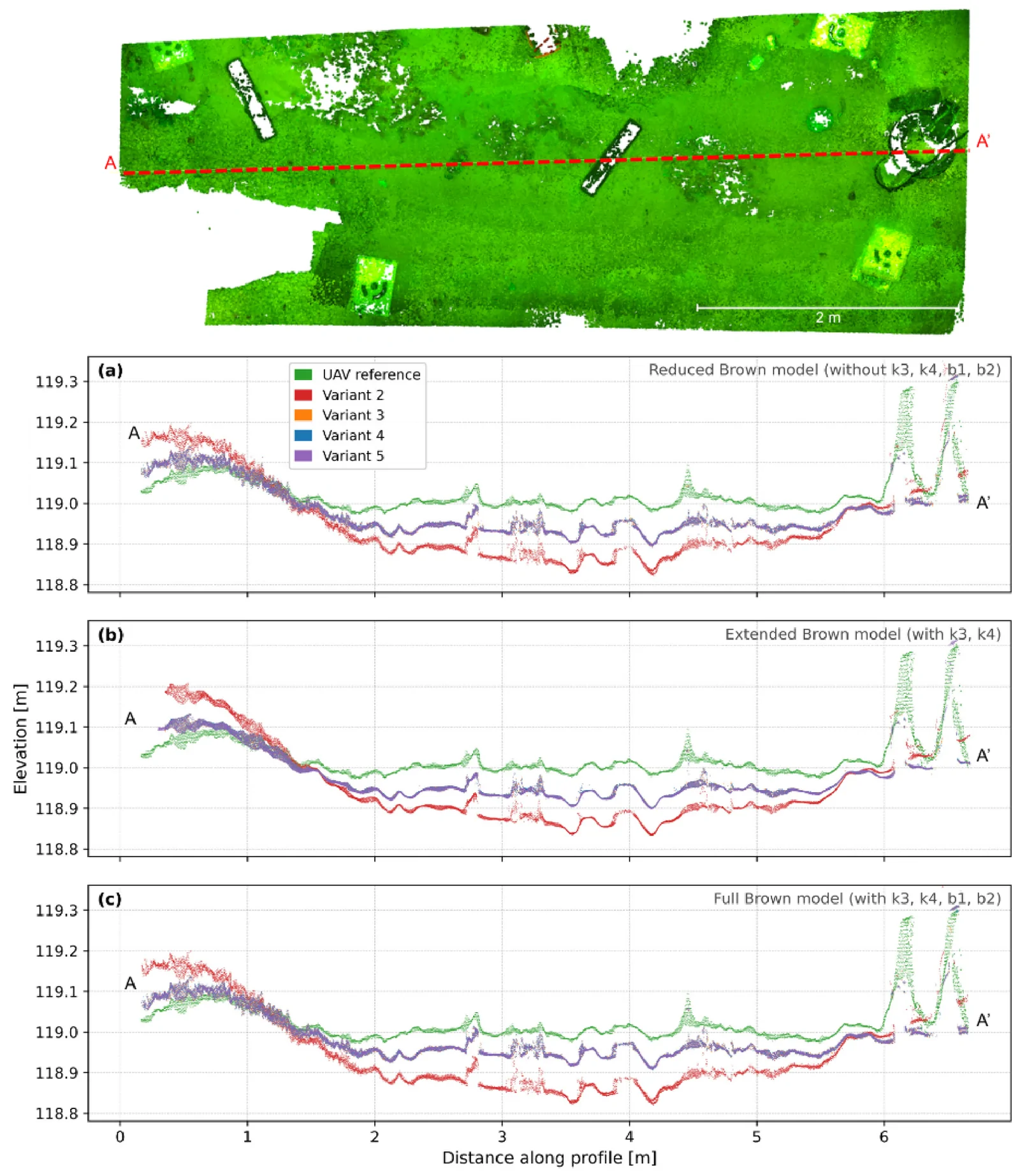
Cross-sectional profiles along transect A–A′ for three Brown model configurations: (**a**) reduced model (without *k*3, *k*4, *b*1, *b*2), (**b**) extended model (with *k*3, *k*4), and (**c**) full model (with *k*3, *k*4, *b*1, *b*2).

Analysis of the cross-sections along section A–A′ (Figure 8) confirms the results in Table 8. In all configurations, Variant 2 consistently underestimates elevation relative to the UAV reference point throughout the profile. Variants 3–5 cluster together. However, across all three configurations, they are systematically shifted approximately 10 mm below the UAV reference. The reduced and extended configurations deliver nearly identical profile distributions. The full configuration shows a slight improvement in the central part of the profile, but the systematic offset relative to the UAV reference remains.

The validation results for the 12 GNSS control points (Table 9) are consistent with the M3C2 versus UAV observations. Variant 2 achieves the highest 3D mean values across all configurations. Variants 3–5 demonstrate high mutual consistency. The systematic components X, Y and Z are close to zero for Variants 3–5, indicating an absence of significant systematic error. The full configuration achieves the lowest mean 3D, standard deviation 3D and maximum 3D values.

Based on all the validation indicators used, the optimal calibration strategy for the Mapir camera in underwater photogrammetric reconstruction is the full configuration combined with Variants 3, 4 or 5. Of these, Variant 5 (in-situ autocalibration) is preferable, as it eliminates the need for additional calibration images and simplifies the data acquisition process in an underwater environment.

## 4. Discussion

Although a wide-angle lens was used, the frame camera model for the GoPro Hero 12 produced lower RMSE reprojection errors under underwater conditions than the fisheye model. Lens distortion is also markedly higher in consumer-grade cameras than in professional systems [44]. The water environment affects the propagation of light in an optical system, leading to changes in the effective parameters of the lens compared to those determined in air. As a result, the calibration parameters estimated underwater differ from those obtained under air conditions [44]. The change in distortion characteristics due to refraction suggests that a simplified frame model may better describe the GoPro lens in an aquatic environment than a fisheye model. This observation is partially consistent with the results of Jarron et al. [72]. The researchers demonstrated the fisheye model’s superiority for the GoPro in the air. This confirms that the optimal camera model depends on the acquisition environment rather than the lens characteristics themselves.

Air calibration (Variant 1) was found to be unsuitable for both cameras. This is because the parameters determined in air do not reflect underwater conditions, due to refraction effects at the water–lens interface and chromatic aberrations. These effects do not occur during air calibration [73]. This finding is supported by a study emphasising the necessity of calibration in the relevant medium [44]. However, it is worth noting that underwater calibration using fixed parameters (Variant 2) produced worse results than optimisation-based approaches. Therefore, the calibration environment alone is insufficient. The ability to adapt parameters to a specific scene is also crucial.

In-situ autocalibration (Variant 5) produced results that were comparable to or better than those of the pre-calibration approaches for both cameras. This is particularly important in practice, as it eliminates the need to take separate underwater images of the calibration panel. This approach is also useful in hard-to-reach environments or when the time available for acquisition is limited.

The optimal configuration of the Brown model parameters depended on which camera was used. The best results with the GoPro camera were obtained using the reduced configuration, whereas the full configuration was more advantageous for the Mapir camera. The observed difference may be partially attributed to differences in the number and density of control points. The GoPro dataset contained approximately 900,000 tie points, whereas the Mapir dataset contained about three times as many. However, it should be emphasised that the quality of bundle adjustment depends not only on the number of control points, but also on their distribution, location accuracy and the number of observations [74]. Using a simplified model may be sufficient to achieve satisfactory results for a dense measurement network. However, adding further parameters could lead to the model being overparameterised and fitted to measurement noise. For the Mapir camera, better results with the full model suggest that these additional parameters more accurately describe the imaging system’s actual distortions. Nevertheless, confirming this relationship requires further research that considers the image network geometry and the distribution of control points.

For the Mapir camera, all configurations demonstrate a consistent shift in reconstruction compared to the UAV reference. This can be seen as an uneven distribution of M3C2 distances on the maps. This pattern is present regardless of how the parameters are configured. Therefore, its source is not the calibration model, but rather other factors. Possible causes include lower resolution in multispectral images, fewer tie points, and the specific optical properties of multispectral filters in water environments. To the best of the authors’ knowledge, this is the first study to use a consumer-grade multispectral camera for underwater photogrammetric reconstruction. This makes a direct comparison with the existing literature impossible. Further research on this topic is required.

The GoPro reconstruction showed a smaller mean signed M3C2 distance relative to the UAV-derived reference cloud than the Mapir reconstruction (6 mm and 38 mm, respectively). These values describe relative agreement with the reference cloud and should not be interpreted as absolute reconstruction accuracies. This can be attributed to the higher resolution of the RGB images, greater detectability of seabed texture features in the visible spectrum, and a higher observation density. Despite these differences, the two cameras complement each other: the GoPro provides more precise geometric data, while the Mapir enables the acquisition of additional spectral information not available to RGB cameras.

The experiment was conducted in a controlled laboratory environment, using a tank with a maximum water depth of 0.5 m. The camera captured images of the bottom from close up and at narrow angles. However, these conditions may differ from those found in deeper bodies of water. Further verification is therefore necessary to determine whether the specified calibration parameter configuration will be equally effective in deeper environments.

Images of the calibration panel were taken from a single distance, and no oblique angles were used. This restricts the geometry of the calibration network, which is a common limitation of underwater environments. This may partly explain the poorer results of Variant 2 and, in the reduced configuration, of Variant 4, which are based on fixed parameters from the calibration panel. Variant 5 does not depend on the panel and produced the smallest or comparable deviations relative to the external reference datasets in most cases, which supports the practical value of in situ self-calibration.

The uncertainty in the final 3D reconstruction resulting from camera calibration parameter uncertainty was not formally assessed. Such an analysis would require the full covariance matrices from the bundle adjustment and their propagation through the photogrammetric workflow. Instead, the results were evaluated using two independent external validation metrics: M3C2 distances relative to the UAV reference point cloud and C2C distances relative to GNSS profiles. The mean values of both metrics are reported with 95% confidence intervals.

Although refraction was not modelled explicitly, its effects were partly absorbed by the Brown model parameters during calibration. For the GoPro, the focal lengths obtained for the underwater variants (V2, V4 and V5) were 1.30–1.50 times higher than the corresponding air-calibrated values, with a median ratio of approximately 1.40. This is close to, although slightly above, the expected magnification of approximately 1.33× for a lens immersed directly in water. However, this agreement does not confirm that refraction was fully accounted for, as the focal length is correlated with the distortion parameters. The differences between calibration variants cannot be explained by refractive magnification alone and may also result from limitations in network geometry and parameter correlations. Future studies should investigate these effects using images acquired at multiple distances and oblique angles, as well as calibration methods that explicitly account for refraction.

Further research should also validate these findings in natural underwater environments at different depths. This will require improved calibration geometry, a larger number of control points, repeated measurement sessions, and formal uncertainty propagation from the calibration parameters to the final 3D reconstruction.

## 5. Conclusions

This paper analyses the impact of calibration methods and Brown model parameter configurations on the accuracy of photogrammetric reconstruction of underwater scenes in a controlled laboratory environment with a maximum water depth of 0.5 m. Two cameras were used in the study: a GoPro Hero 12 RGB camera and a Mapir Survey3W multispectral camera. The results showed that neither camera model was suitable for air calibration under the test conditions. This suggests that parameters determined in the air cannot be transferred to the underwater environment. In-situ autocalibration produced M3C2 and C2C deviations comparable to or smaller than those obtained using preliminary calibration. This is advantageous as it eliminates the need to take separate underwater photos of the calibration panel.

For the GoPro Hero 12, better underwater results were obtained using the frame model than the fisheye model in laboratory conditions, despite the latter’s wide-angle lens. This demonstrates that the underwater environment can affect the optical properties of the lens. In practice, this means that a camera model selected for aerial use may not be the best choice for underwater measurements. Nevertheless, generalising this conclusion to other depths and environments requires further verification. For the GoPro camera, the reduced configuration proved most advantageous. Adding higher-order terms did not improve agreement with the external reference datasets under the tested conditions, and the extended variant exhibited systematic geometric distortions. However, the full variant showed better validation results for the Mapir camera. These results provide a reference for further studies in natural underwater environments and at greater depths. Repeating the measurements in separate sessions would help assess the repeatability and statistical stability of the calibration strategies.

## Figures and Tables

**Table 1:** Camera system specifications.

Parameter	Camera 1	Camera 2
Camera model	GoPro HERO12 Black	Mapir Survey3W (RGN)
Spectral bands	RGB	RGN
Sensor size	1/1.9″	1/2.3″ CMOS
Sensor resolution [MPx]	27	12
Focal length [mm]	3	3
Image format	JPG	JPG
Image resolution [pix]	5568 × 4872	4000 × 3000
Lens type	Fisheye	Frame
Housing	none	with flat port

**Table 2:** Distribution of residual errors.

	Variant 1	Variant 2	Variant 3	Variant 4	Variant 5
Fisheye camera model					
RMSE [pix]	366	1.93	0.89	1.49	0.98
RMSE max. [pix]	1400	17.10	94.42	13.60	9.42
Frame camera model					
RMSE [pix]	20.40	0.37	1.11	0.31	0.27
RMSE max. [pix]	378	4.30	53.70	2.59	1.77

**Table 3:** GCP errors for the GoPro-based processing variants using the frame camera models with three parameter configurations.

	Variant 1	Variant 2	Variant 3	Variant 4	Variant 5
Reduced: without *k*3, *k*4, *b*1, *b*2					
X error [mm]	2219	7	4	4	4
Y error [mm]	241	7	3	5	3
Z error [mm]	413	33	12	34	11
Total error [mm]	2270	35	13	35	12
Image error [pix]	7.036	0.444	0.545	0.396	0.710
Extended: without *b*1, *b*2					
X error [mm]	2015	10	8	5	5
Y error [mm]	405	7	2	2	2
Z error [mm]	497	32	8	8	7
Total error [mm]	2115	34	10	10	9
Image error [pix]	50.142	1.330	1.229	1.205	1.224
Full: with *k*3, *k*4, *b*1, *b*2					
X error [mm]	2256	5	5	4	4
Y error [mm]	283	4	5	2	2
Z error [mm]	364	38	28	1	1
Total error [mm]	2302	39	28	5	5
Image error [pix]	8.136	0.411	0.499	0.365	0.365

**Table 4:** M3C2 distance statistics for the GoPro-based processing variants relative to the reference UAV image-based bathymetric point cloud.

	Variant 1	Variant 2	Variant 3	Variant 4	Variant 5
Reduced: without *k*3, *k*4, *b*1, *b*2					
Mean 3D [mm]	-	61	4	−22	6
Median 3D [mm]	-	73	2	−28	2
Std. dev. 3D [mm]	-	75	33	60	29
P95 3D [mm]	-	151	56	98	43
<20 mm [%]	-	11.75	69.62	21.00	77.52
<50 mm [%]	-	30.63	93.69	50.47	95.98
<100 mm [%]	-	61.29	98.17	95.54	98.40
95% CI [mm]	-	60.6–63.0	3.3–4.6	−23.2–−20.8	4.9–6.1
Extended: without *b*1, *b*2					
Mean [mm]	-	57	30	30	29
Median [mm]	-	72	33	34	32
Std. dev. [mm]	-	72	34	37	33
P95 [mm]	-	142	66	69	65
<20 mm [%]	-	11.83	28.84	25.70	29.83
<50 mm [%]	-	30.91	78.06	74.74	82.10
<100 mm [%]	-	63.11	97.80	97.64	97.88
95% CI [mm]	-	56.5–59.3	28.8–30.1	29.8–31.2	28.5–29.8
Full: with *k*3, *k*4, *b*1, *b*2					
Mean 3D [mm]	-	67	53	21	21
Median 3D [mm]	-	78	65	21	21
Std. dev. 3D [mm]	-	80	69	28	28
P95 3D [mm]	-	163	136	55	56
<20 mm [%]	-	11.55	12.81	44.04	43.87
<50 mm [%]	-	29.83	33.02	93.90	93.85
<100 mm [%]	-	56.96	71.96	98.32	98.31
95% CI [mm]	-	65.7–69.0	51.4–54.0	20.1–21.1	20.3–21.3

**Table 5:** Statistics of signed C2C coordinate differences in the X, Y, and Z components, and absolute 3D C2C distances, for the GoPro-based processing variants relative to GNSS-measured profiles.

	Variant 1	Variant 2	Variant 3	Variant 4	Variant 5
Reduced: without *k*3, *k*4, *b*1, *b*2					
Mean X [mm]	-	5	−1	2	−2
Mean Y [mm]	-	2	0	−1	1
Mean Z [mm]	-	15	−4	−15	−4
Mean 3D [mm]	-	65	13	52	16
Median 3D [mm]	-	60	11	56	16
Std. dev. 3D [mm]	-	32	11	26	8
Max 3D [mm]	-	129	55	107	33
95% CI [mm]	-	52.3–77.4	8.9–17.1	42.1–62.5	12.6–18.5
Extended: without *b*1, *b*2					
Mean X [mm]	-	4	1	1	2
Mean Y [mm]	-	3	−1	−2	−2
Mean Z [mm]	-	14	6	6	6
Mean 3D [mm]	-	64	22	22	20
Median 3D [mm]	-	58	19	20	19
Std. dev. 3D [mm]	-	32	14	13	14
Max 3D [mm]	-	126	52	53	50
95% CI [mm]	-	51.2–75.8	16.2–27.3	17.3–27.4	15.0–26.0
Full: with *k*3, *k*4, *b*1, *b*2					
Mean X [mm]	-	1	3	0	0
Mean Y [mm]	-	12	−1	−1	0
Mean Z [mm]	-	13	12	3	2
Mean 3D [mm]	-	73	55	12	12
Median 3D [mm]	-	72	54	10	10
Std. dev. 3D [mm]	-	41	27	7	7
Max 3D [mm]	-	145	110	30	30
95% CI [mm]	-	57.5–89.0	44.5–65.8	8.9–14.6	8.8–14.7

**Table 6:** Distribution of residual errors.

	Variant 1	Variant 2	Variant 3	Variant 4	Variant 5
Frame camera model					
RMSE [pix]	49.3	0.46	0.32	0.32	0.32
RMSE max. [pix]	2450	3.96	2.99	3.00	2.99

**Table 7:** GCP errors for the Mapir-based processing variants using the frame camera models with three parameter configurations.

	Variant 1	Variant 2	Variant 3	Variant 4	Variant 5
Reduced: without *k*3, *k*4, *b*1, *b*2					
X error [mm]	367	6	7	7	7
Y error [mm]	337	4	3	3	3
Z error [mm]	395	27	4	7	4
Total error [mm]	636	28	10	10	10
Image error [pix]	11.582	1.159	1.020	1.023	1.019
Extended: without *b*1, *b*2					
X error [mm]	1113	5	7	7	7
Y error [mm]	496	6	3	3	3
Z error [mm]	219	20	7	7	7
Total error [mm]	1238	22	10	10	11
Image error [pix]	10.713	1.050	1.012	1.012	0.991
Full: with *k*3, *k*4, *b*1, *b*2					
X error [mm]	369	7	7	7	7
Y error [mm]	252	5	3	3	3
Z error [mm]	313	34	3	3	3
Total error [mm]	546	35	9	9	9
Image error [pix]	10.813	1.158	0.982	0.982	0.982

**Table 8:** M3C2 distance statistics for the Mapir-based processing variants relative to the reference UAV image-based bathymetric point cloud.

	Variant 1	Variant 2	Variant 3	Variant 4	Variant 5
Reduced: without *k*3, *k*4, *b*1, *b*2					
Mean 3D [mm]	-	63	42	42	42
Median 3D [mm]	-	79	44	44	44
Std. dev. 3D [mm]	-	67	40	39	40
P95 3D [mm]	-	144	111	110	111
<20 mm [%]	-	11.58	22.84	22.97	22.90
<50 mm [%]	-	29.50	56.99	57.25	57.01
<100 mm [%]	-	61.96	92.74	92.89	92.72
95% CI [mm]	-	61.7–64.4	41.5–43.0	41.4–42.9	41.8–43.4
Extended: without *b*1, *b*2					
Mean 3D [mm]	-	62	43	43	43
Median 3D [mm]	-	78	44	44	44
Std. dev. 3D [mm]	-	67	40	40	40
P95 3D [mm]	-	141	112	112	113
<20 mm [%]	-	11.30	21.99	20.06	22.87
<50 mm [%]	-	29.23	56.95	56.99	56.96
<100 mm [%]	-	62.74	92.35	92.32	92.34
95% CI [mm]	-	60.7–63.3	42.4–44.0	42.1–43.6	41.8–43.3
Full: with *k*3, *k*4, *b*1, *b*2					
Mean 3D [mm]	-	63	38	38	38
Median 3D [mm]	-	79	37	37	37
Std. dev. 3D [mm]	-	68	36	35	36
P95 3D [mm]	-	146	105	105	106
<20 mm [%]	-	11.18	27.49	27.37	27.46
<50 mm [%]	-	29.04	69.13	69.09	69.18
<100 mm [%]	-	62.63	93.97	93.96	93.95
95% CI [mm]	-	61.2–64.0	37.0–38.4	37.0–38.4	37.1–38.5

**Table 9:** Statistics of signed C2C coordinate differences in the X, Y, and Z components and absolute 3D C2C distances for 12 check points relative to GNSS measured profiles.

Statistic	Variant 1	Variant 2	Variant 3	Variant 4	Variant 5
Reduced: without *k*3, *k*4, *b*1, *b*2					
Mean X [mm]	-	4	0	0	0
Mean Y [mm]	-	−4	−1	−1	−3
Mean Z [mm]	-	5	−1	0	−1
Mean 3D [mm]	-	69	28	28	29
Median 3D [mm]	-	76	28	26	28
Std. dev. 3D [mm]	-	29	11	12	12
Max 3D [mm]	-	104	47	48	50
95% CI [mm]	-	51.5–87.8	20.4–35.1	20.1–35.2	20.7–36.3
Extended: without *b*1, *b*2					
Mean X [mm]	-	−2	−1	0	−3
Mean Y [mm]	-	1	−2	−2	−3
Mean Z [mm]	-	8	0	0	0
Mean 3D [mm]	-	69	27	27	28
Median 3D [mm]	-	78	26	26	27
Std. dev. 3D [mm]	-	28	12	12	12
Max 3D [mm]	-	101	47	48	49
95% CI [mm]	-	50.7–86.9	19.6–34.6	19.9–34.5	20.4–35.6
Full: with *k*3, *k*4, *b*1, *b*2					
Mean X [mm]	-	2	−1	−2	0
Mean Y [mm]	-	−3	−1	−2	−1
Mean Z [mm]	-	2	−1	0	−1
Mean 3D [mm]	-	74	21	21	21
Median 3D [mm]	-	80	20	21	21
Std. dev. 3D [mm]	-	32	10	10	10
Max 3D [mm]	-	122	39	39	38
95% CI [mm]	-	54.3–94.5	14.9–27.3	15.1–27.1	14.7–27.3

## Data Availability

Dataset available on request from the authors.

## Appendix A. Supplement to Camera Model Selection

**Table A1:** Camera calibration parameters estimated for the GoPro-based processing variants using the fisheye and frame camera models.

Parameter	Variant 1	Variant 2	Variant 3	Variant 4	Variant 5
Fisheye camera model					
*F*	2448.0539	3623.8164	6133.5466	4844.2349	6134.2804
cx	−1.4255	−11.6791	−28.1825	−21.5053	−28.1797
cy	6.7212	39.3322	−8.2086	10.1610	−8.1957
k1	0.0408	0.0759	−0.1263	−0.0042	−0.1263
k2	0.0024	0.5132	1.8063	0.9256	1.8070
p1	−0.000940	0.001204	0.005070	0.002911	0.005070
p2	−0.001165	−0.003691	0.000130	−0.001862	0.00012
Frame camera model					
*F*	2316.0558	3476.9087	3788.5335	3333.2806	3228.3398
cx	−42.1722	−11.7566	−9.6228	−4.2388	−4.0844
cy	39.9962	15.4456	−1.7365	−0.1024	−2.2104
k1	−0.1660	−0.1268	−0.1359	−0.1001	−0.0989
k2	0.0173	0.1238	0.1775	0.1092	0.0943
p1	−0.000233	0.000777	0.000748	−0.000002	−0.000228
p2	−0.000450	−0.002238	−0.000234	−0.000312	−0.000210

## Appendix B. Supplement to Higher-Order Radial Distortion Analysis

**Table A2:** Camera calibration parameters estimated for the GoPro-based processing variants using the frame camera models.

Parameter	Variant 1	Variant 2	Variant 3	Variant 4	Variant 5
Reduced: without *k*3, *k*4, *b*1, *b*2					
*F*	2316.0558	3476.9087	3788.5335	3333.2806	3228.3398
cx	−42.1722	−11.7566	−9.6228	−4.2388	−4.0844
cy	39.9962	15.4456	−1.7365	−0.1024	−2.2104
k1	−0.1660	−0.1268	−0.1359	−0.1001	−0.0989
k2	0.0173	0.1238	0.1775	0.1092	0.0943
p1	−0.000233	0.000777	0.000748	−0.000002	−0.000228
p2	−0.000450	−0.002238	−0.000234	−0.000312	−0.000210
Extended: without *b*1, *b*2					
*F*	2395.6890	3482.6573	3250.0055	3425.7069	3249.2785
cx	−18.6345	−12.2593	−4.5652	−6.5458	−4.5335
cy	8.96538	13.5767	−4.5775	−5.8272	−4.1169
k1	−0.2336	−0.1722	−0.1367	−0.1587	−0.1364
k2	0.0611	0.31524	0.2181	0.3015	0.2178
k3	−0.0091	−0.3023	−0.1752	−0.2972	−0.1749
k4	0.0005	0.1585	0.0826	0.1585	0.0824
p1	0.000331	0.000697	−0.000180	−0.000181	−0.000180
p2	−0.000427	−0.002243	−0.000119	−0.000051	−0.000139
Full: with *k*3, *k*4, *b*1, *b*2					
*F*	2386.6265	3110.9217	3711.3140	3339.3252	3338.8680
cx	−19.4590	−12.1362	−9.46114	−5.42945	−5.4256
cy	−4.1085	−28.2388	−4.23328	−2.90658	−2.9116
k1	−0.2330	−0.137091	−0.177139	−0.137376	−0.1374
k2	0.0605	0.198812	0.339504	0.223152	0.2230
k3	−0.0089	−0.1521	−0.3291	−0.1766	−0.1765
k4	0.000515	0.0632239	0.198478	0.0885656	0.0885
p1	0.000217	0.000583	0.000709	0.000127	0.000122
p2	0.000365	−0.001115	−0.000118	−0.000177	−0.000177
b1	5.6729	12.9151	2.1478	−0.0756	−0.0664
b2	9.8693	2.2267	2.4353	1.4288	1.4298

**Table A3:** Camera calibration parameters estimated for the variants of processing based on the photos from Mapir.

Parameter	Variant 1	Variant 2	Variant 3	Variant 4	Variant 5
Reduced: without *k*3, *k*4, *b*1, *b*2					
*F*	2289.5976	2981.8726	2783.7258	2783.9608	2783.7101
cx	−47.5924	−15.4894	−11.7922	−11.7897	−11.7923
cy	112.138	65.438	44.6864	44.878	44.6732
k1	0.0154	0.5967	0.5103	0.5104	0.5103
k2	−0.0510	−0.2339	−0.1341	−0.1341	−0.1341
p1	−0.001379	0.016708	0.017476	0.017488	0.017475
p2	0.001702	−0.010385	−0.009149	−0.009110	−0.009151
Extended: without *b*1, *b*2					
*F*	2283.3533	2971.5382	2782.7409	2782.6529	2782.7839
cx	−47.6358	−17.0956	−14.8736	−14.8807	−15.1763
cy	113.5490	69.1015	50.5391	50.4958	50.5708
k1	0.0466	0.7918	0.5756	0.5756	0.5758
k2	−0.1185	−1.7866	−0.5555	−0.5555	−0.5571
k3	0.0426	4.9280	1.1119	1.1120	1.1148
k4	−0.0002	−5.3187	−0.9956	−0.9958	−0.9961
p1	−0.001612	0.01624	0.016593	0.016594	0.016475
p2	0.001804	−0.009828	−0.008151	−0.008157	−0.008154
Full: with *k*3, *k*4, *b*1, *b*2					
*F*	2216.1555	2950.3120	2799.5646	2799.5640	2799.5648
cx	−53.7790	16.6982	−15.5642	−15.5631	−15.5646
cy	101.9520	44.9381	64.8810	64.8800	64.8812
k1	0.0492	0.7372	0.5860	0.5860	0.5860
k2	−0.1283	−1.3767	−0.5811	−0.5811	−0.5811
k3	0.0627	3.4796	1.1925	1.1925	1.1925
k4	−0.0101	−3.5668	−1.0852	−1.0852	−1.0852
p1	−0.000526	0.021221	0.016492	0.016492	0.016492
p2	0.001485	−0.013675	−0.005200	−0.005200	−0.005200
b1	6.8896	−3.7054	2.9767	2.9762	2.9769
b2	7.7942	−15.5204	2.1477	2.1467	2.1480
